# Effect of subinhibitory exposure to quaternary ammonium compounds on the ciprofloxacin susceptibility of *Escherichia coli* strains in animal husbandry

**DOI:** 10.1186/s12866-020-01818-3

**Published:** 2020-06-11

**Authors:** H. Maertens, K. Demeyere, K. De Reu, J. Dewulf, D. Vanhauteghem, E. Van Coillie, E. Meyer

**Affiliations:** 1 Flanders Research Institute for Agriculture, Fisheries and Food (ILVO), Technology and Food Science Unit, Brusselsesteenweg 370, 9090 Melle, Belgium; 2 Veterinary Biochemistry Unit, Department of Pharmacology, Toxicology and Biochemistry, Faculty of Veterinary Medicine, Ghent University, Salisburylaan 133, 9820 Merelbeke, Belgium; 3 Veterinary Epidemiology Unit, Department of Reproduction, Obstetrics and Herd Health, Faculty of Veterinary Medicine, Ghent University, Salisburylaan 133, 9820 Merelbeke, Belgium

**Keywords:** Biocide exposure, Antibiotic susceptibility, Viability, Culturability, Benzalkonium chloride, Ciprofloxacin, *Escherichia coli*, Flow cytometry, Quaternary ammonium compound

## Abstract

**Background:**

Quaternary ammonium compound based disinfectants are commonly used in pig and poultry husbandry to maintain farm hygiene. However, studies have shown that subinhibitory concentrations of these disinfectants may increase antibiotic resistance. Investigation of antibiotic susceptibility is usually assessed via the microbroth dilution method, although this conventional culture-based technique only provides information on the bacteriostatic activity of an antimicrobial agent. Therefore, experiments were performed to investigate the effect of prior benzalkonium chloride (BKC) exposure on the viability of subsequent ciprofloxacin (CIP) treated *Escherichia coli.*

**Results:**

Following CIP treatment, bacterial cell counts were significantly higher after exposure to a subinhibitory BKC concentration than without BKC exposure. The flow cytometric results suggested a BKC-dependent onset of membrane damage and loss of membrane potential.

**Conclusion:**

Our results indicate a lower bactericidal effect of CIP treatment on BKC-exposed *E. coli* isolates compared to unexposed *E. coli* isolates.

## Background

Biocides are chemical agents that inactivate (micro)organisms [1], including disinfectants, preservatives, pest control agents and other biocidal products. Depending on their target, disinfectants can be classified into following types: products for human hygiene; disinfectants and algaecides not intended for direct application to humans or animals; and products for veterinary hygiene, food and feed areas, and drinking water (BPR, Regulation (EU) 528/2012).

Commercial farm disinfectants are often composed of two or more active disinfectant components with different targets and modes of action. Quaternary ammonium compounds (QACs) are among the most widely used active components in farm disinfectants [2]. Their use for veterinary hygiene purposes plays a crucial role in the prevention of spreading bacterial infections within and between herds, an important aspect of on-farm biosecurity. With the global need to reduce the emergence of antibiotic resistance, the application of adequate cleaning and disinfection has become increasingly important. Disinfectants effectively kill bacteria when applied correctly, but their inappropriate use (e.g., dilution in rinsing water or inactivation due to residual organic matter) can lead to a reduced bactericidal effect. In such instances, bacteria are exposed to subinhibitory concentrations of disinfectants.

A common method to investigate the effect of antimicrobial components on bacterial growth is based on defining the minimal inhibitory concentration (MIC) via broth dilution. This method defines the lowest concentration of an antimicrobial component at which no bacterial growth is visually observed. Furthermore, extended and sequential growth at subinhibitory concentrations of disinfectants has been shown to lead to an increase in antibiotic MICs in vitro [3, 4]. However, neither study provides information about how the bactericidal activity of the antibiotics becomes modified due to bacterial growth in subinhibitory concentrations of disinfectants.

Therefore, the main objective of the current study was to evaluate the impact of prior exposure to subinhibitory QAC concentrations on the growth of antibiotic-treated *E. coli* isolates via plate count analysis. Nevertheless, it should be emphasized that the traditional culture-based or growth-dependent counting methods could underestimate the tolerance of microorganisms to disinfectants. It is indeed known that bacteria can respond to external stress conditions by reverting into a viable but non-culturable (VBNC) state [5, 6]. While in this dormant state, bacteria can no longer be cultivated, although they are still viable [7]. A complementary growth-independent technique is flow cytometry (FCM) which can provide additional information about the physiological properties of the bacterial population via the use of different fluorescent stains [8, 9]. FCM is a powerful tool to assess the effects of antibacterial agents on bacterial viability [10–12]. Such analysis of bacterial populations at single-cell level is also highly informative regarding population heterogeneity. Selection of appropriate fluorescence markers makes it possible to evaluate the desired phenotypic and physiological characteristics of individual cells. Studies of the microbial culturability and viability after either antibiotic treatment [12] or disinfection procedures [13, 14], or investigating loss of bacterial viability after exposure to disinfectants [15] are scarce. To our knowledge, no studies have been performed that describe the effect of prior QAC exposure on the bacterial viability response to antibiotic treatment*.* Therefore, we measured the viability state of porcine and avian *E. coli* animal husbandry isolates to better understand bacterial resistance behaviour after exposure to the QAC biocide BKC.

## Results

### Bacterial plate counts

**Fig. 1:**
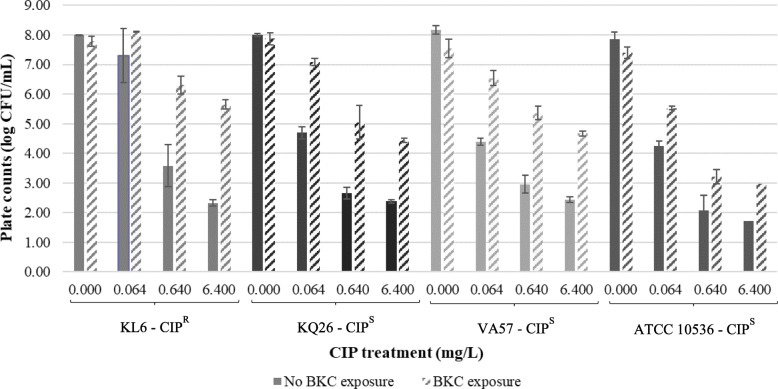
Plate counts (log CFU/mL) for each ciprofloxacin-susceptible (CIP^S^) and ciprofloxacin-resistant (CIP^R^) *Escherichia coli* strain after CIP treatment with and without prior exposure to benzalkonium chloride (BKC). Data are expressed as means ± standard deviations of triplicate experiments

The effect of prior exposure to subinhibitory concentrations of BKC on the number of culturable bacteria after CIP treatment at different concentrations was first investigated by plate counting. Mean values of the bacterial counts (log colony forming units (CFU)/mL) of each tested *E. coli* isolate for each CIP concentration are given in Fig. 1. Results of the uni- and multivariable regression model are listed in Table S1 (see Additional File 1). All tested variables were significant and were thus added to the multivariable model

Overall, the mean bacterial counts were significantly higher after CIP treatment with exposure to BKC than without BKC exposure (*P* < 0.001). However, the BKC effect was minimal for the CIP-resistant strain at a CIP concentration lower than the MIC (i.e., 0.064 mg/L).

Furthermore, a significant association (*P* < 0.001) was found between CIP treatment and the bacterial counts with lower *E. coli* counts upon increasing CIP concentrations. Even when treated with CIP concentrations of > 10 to > 100 times above the MIC (i.e. 0.640 and 6.400 mg/L CIP), countable numbers of bacteria were found for all *E. coli* isolates, including the three CIP-susceptible isolates. Important to note is that the inoculum size of the standardized method to determine a MIC value is 1–5 × 10^5^ CFU/mL while in the current study an inoculum size of 1–5 × 10^8^ CFU/mL was used.

Significant differences (*P* < 0.001) in the mean CFU/mL values were also observed between the one CIP-resistant and the three CIP-susceptible isolates. Especially for concentrations of 0.064 and 0.640 mg/L CIP, higher bacterial counts were obtained for the CIP-resistant isolate compared to the mean bacterial counts for the CIP-susceptible isolates. Moreover, for the most susceptible isolate, the lowest bacterial counts were observed as well as a more distinct decrease in CFU/mL by increasing CIP concentrations.

### Flow cytometric assessment of bacterial membrane potential and integrity

Parallel to the evaluation of bacterial culturability via plate counting, the growth-independent membrane potential and integrity were also analysed by dual staining FCM. The membrane potential was assessed using bis-(1,3-dibutylbarbituric acid) trimethine oxonol (DiBAC_4_ (3)). This dye enters depolarised cells and binds to lipid-containing intracellular components. The second dye used was Propidium Iodide (PI) to measure the membrane integrity. This dye stains dead cells by intercalating with DNA as it can only enter membrane damaged cells.

Three bacterial subpopulations could thus be distinguished: the membrane-intact live bacteria, the partly membrane-damaged intermediate bacteria and the irreversibly membrane-damaged dead bacteria with complete loss of membrane potential. FCM results and illustrative dot plots of both live and dead controls are provided in Table S2 and Figure S1, respectively (see Additional Files 2 and 3). It should be noted that the percentage of the live subpopulation in the controls approached 100%, indicating that sonication did not cause a subsequent reduction in microbial viability.

**Fig. 2:**
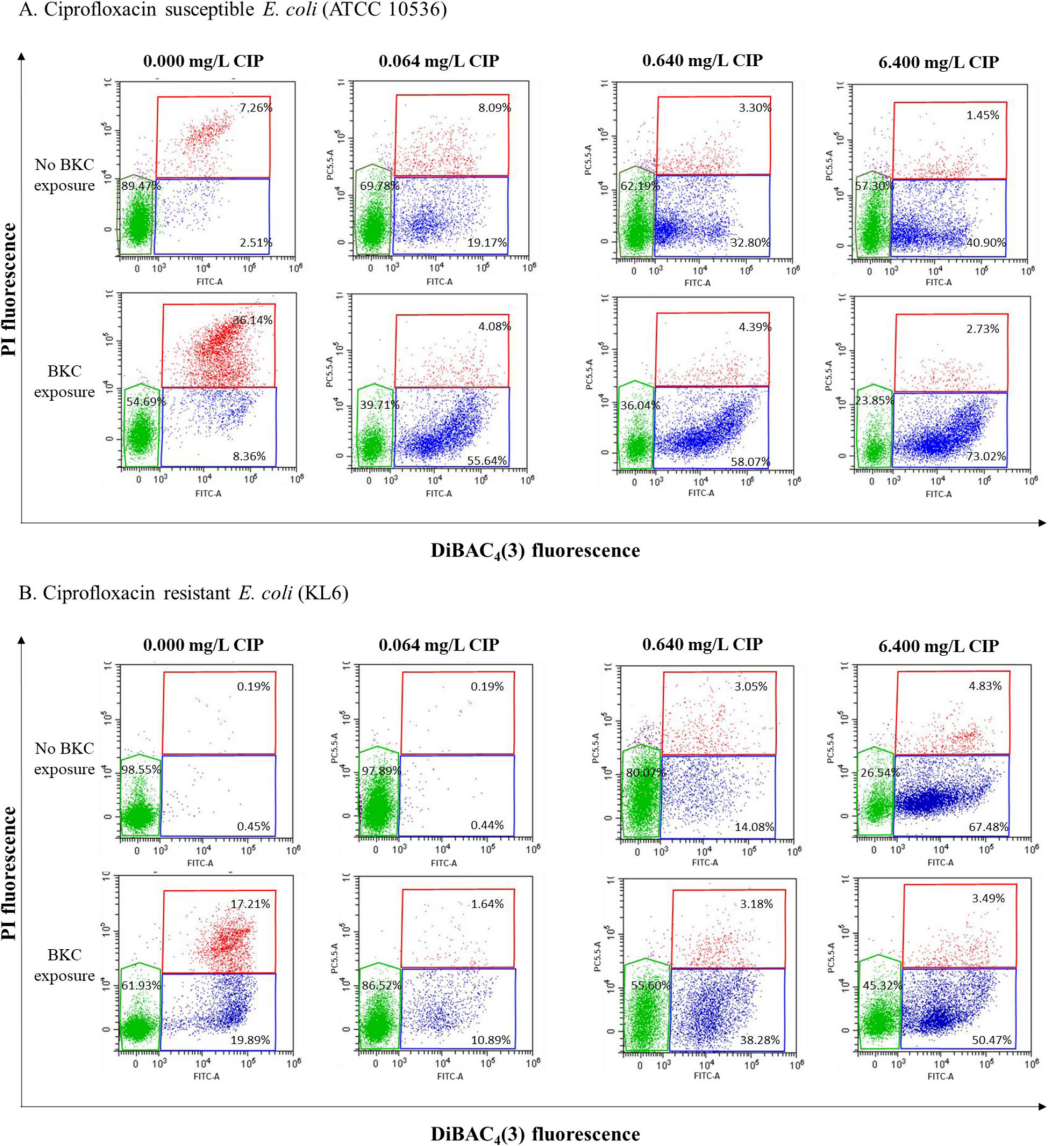
Flow cytometric DIBAC_4_ (3) (FITC-A)/PI (PC5.5A) dot plots representing *Escherichia coli* viability through membrane potential and membrane integrity of a ciprofloxacin (CIP)-susceptible (**a**) and CIP-resistant (**b**) *Escherichia coli* strain after treatment with three increasing CIP concentrations either with or without benzalkonium chloride (BKC) exposure. The green region corresponds to the live subpopulation with an intact membrane and normal membrane potential, the blue region corresponds to the intermediate subpopulation with different degrees of compromised membranes and loss of membrane potential, the red region corresponds to the dead subpopulation with irreversibly damaged membrane and complete loss of membrane potential. Percentages are the mean of triplicate experiments

Results of uni- and multivariable models of *E. coli* membrane potential and integrity are presented in Table S3 (see Additional File 4). Because the three bacterial subpopulations are cumulative, only the live and intermediate subpopulations were evaluated, as these two subpopulations provide the most valuable information for the current study.

FCM results showed a clear shift from the live to the intermediate subpopulation following CIP treatment with BKC exposure in most (8 out of 12) cases (Table 1). In our multivariable model, exposure of the *E. coli* isolates to BKC resulted in a significant increase of intermediate bacteria (*P* = 0.007) whereas a concomitant significant decrease was found for the live subpopulation (*P* = 0.035).

**Table 1:** Viability (%) of each *Escherichia coli* isolate after different antibiotic treatments with and without exposure to benzalkonium chloride, expressed as mean +/− standard deviation

***E. coli*** isolate - origin	MIC (mg/L CIP)	CIP treatment (mg/L)	Subpopulation	No BKC exposure			BKC exposure		
KL6; Poultry	0.120; Resistant (non-WT)	0.000	Live	98.44	±	0.51	61.40	±	2.10
			Intermediate	0.35	±	0.17	20.71	±	0.79
			Dead	0.17	±	0.04	16.89	±	1.64
		0.064	Live	97.15	±	0.82	87.57	±	0.93
			Intermediate	1.02	±	0.55	9.77	±	1.02
			Dead	0.38	±	0.19	1.54	±	0.77
		0.640	Live	76.03	±	3.52	58.75	±	3.20
			Intermediate	18.73	±	4.12	35.67	±	2.58
			Dead	2.97	±	0.34	2.94	±	0.24
		6.400	Live	25.80	±	3.44	49.32	±	3.57
			Intermediate	68.14	±	2.37	44.96	±	4.84
			Dead	5.12	±	1.27	4.38	±	0.77
KQ26; Poultry	0.030; Susceptible* (WT)	0.000	Live	97.03	±	0.30	79.47	±	2.08
			Intermediate	0.95	±	0.18	4.17	±	0.58
			Dead	1.62	±	0.28	15.70	±	1.38
		0.064	Live	51.55	±	0.88	78.27	±	2.95
			Intermediate	42.48	±	1.32	17.34	±	2.13
			Dead	4.95	±	0.41	2.17	±	0.16
		0.640	Live	64.12	±	2.43	63.73	±	5.40
			Intermediate	30.96	±	1.64	31.46	±	5.65
			Dead	3.57	±	0.64	3.48	±	0.12
		6.400	Live	83.16	±	1.60	70.69	±	1.51
			Intermediate	12.91	±	1.30	26.86	±	1.50
			Dead	2.69	±	0.24	1.52	±	0.19
VA57; Pork	0.030; Susceptible* (WT)	0.000	Live	93.97	±	0.30	50.50	±	4.41
			Intermediate	1.37	±	0.02	9.88	±	0.66
			Dead	3.93	±	0.31	38.66	±	4.93
		0.064	Live	51.75	±	2.60	53.87	±	9.77
			Intermediate	34.45	±	3.60	35.97	±	7.70
			Dead	12.68	±	3.32	8.71	±	2.47
		0.640	Live	69.72	±	2.09	51.10	±	3.74
			Intermediate	14.59	±	0.63	40.18	±	2.64
			Dead	13.93	±	1.33	7.71	±	1.42
		6.400	Live	67.12	±	7.49	49.07	±	7.07
			Intermediate	26.31	±	5.79	44.51	±	7.12
			Dead	5.59	±	1.75	5.27	±	0.05
ATCC 10536	≤ 0.015; Susceptible* (WT)	0.000	Live	88.62	±	2.29	54.84	±	2.09
			Intermediate	2.74	±	0.22	6.76	±	1.39
			Dead	7.89	±	2.09	37.07	±	2.04
		0.064	Live	67.56	±	3.68	41.25	±	1.33
			Intermediate	22.19	±	3.79	54.12	±	2.47
			Dead	7.67	±	0.50	4.07	±	0.27
		0.640	Live	55.73	±	6.17	39.37	±	2.91
			Intermediate	38.62	±	7.95	56.31	±	2.47
			Dead	4.42	±	1.44	3.66	±	0.65
		6.400	Live	52.92	±	6.96	27.46	±	4.16
			Intermediate	44.05	±	5.56	67.88	±	4.45
			Dead	2.34	±	1.15	4.06	±	2.69

*MIC* Minimal Inhibitory Concentration, *CIP* ciprofloxacin, *WT* wild type; ^*^susceptible/resistant to CIP, defined by the epidemiologic cutoff value (ECOFF, 0.064 mg/L CIP) separating the bacterial population into those without (wild type; <= 0.064 mg/L CIP ), and those with acquired resistance (non-wild type; > 0.064 mg/L CIP)

Furthermore, even with the small number (*n* = 4, including one ATCC reference strain) of *E. coli* isolates used in this study, a clear difference between the one CIP-resistant and the three CIP-susceptible isolates was observed when treated with a CIP concentration below the MIC (i.e. 0.064 mg/L). In contrast to the CIP-susceptible isolates, an intact bacterial membrane integrity and potential was shown for the CIP-resistant isolate. Representative dot plots of a CIP-susceptible compared to the CIP-resistant *E. coli* strain with and without exposure to BKC for each CIP concentration are presented in Fig. 2. Nevertheless, there was no significant association between the CIP-resistance profile and the live and intermediate subpopulations in the univariable model.

Although the variables ‘CIP treatment’ and ‘origin’ were significantly associated with both live (*P* = 0.009 and 0.001, respectively) and intermediate subpopulations (*P* < 0.001 and *P* = 0.002, respectively), no trend between the CIP-resistant and CIP-susceptible isolates after treatment with increasing concentrations of CIP could be found.

## Discussion

Several studies investigated the changes in antibiotic susceptibility of *E. coli* after exposure to a biocide using MIC determination [3, 4]. Nevertheless, these reported MIC values only provide information on the inhibition of the bacterial growth capacity but do not evaluate the bactericidal capacity of the antimicrobial agent [16, 17]. Since plate counts assess both of these related parameters, this was used as the main growth-dependent method in the current study. While conventional growth-dependent methods typically assess bacterial culturability, complementary growth-independent methods like FCM are increasingly used for viability assessment [12, 13, 15].

Therefore, the main aim of our study was to investigate changes in culturability via plate counts and changes in viability via FCM of CIP-treated avian and porcine *E. coli* isolates following their exposure to subinhibitory QAC concentrations versus non-QAC exposed CIP-treated bacteria.

Based on our plate count data, the first key finding was the lower bactericidal effect of CIP treatment on BKC-exposed *E. coli* isolates compared to unexposed *E. coli* isolates. As for the growth-independent bacterial viability assessment, FCM also revealed a clear shift from the live to the intermediate bacterial subpopulation following CIP treatment with BKC exposure. The QAC biocide BKC contains a positively charged quaternary nitrogen which interacts with the phospholipids within the bacterial membrane. These interactions increase the membrane surface pressure [18] and disrupt the charge distribution [19]. The resulting partly depleted membrane potential could explain the decrease in the *E. coli* viability state from live to intermediate as observed by the DiBAC_4_ (3) flow cytometrical staining. In addition, changes in membrane potential - e.g., those resulting from BKC exposure - are considered as an indicator of injury which could be reversible, although this reversibility depends on the organism, the cause of injury and the environment [20]. On the other hand, these changes in membrane potential may also reflect a (semi-)dormant persistent state of the BKC-exposed cells [7]. A decreasing membrane potential has been previously described in cells treated with CIP [21, 22] and its relation to the bacterial repair SOS-system has been confirmed which is also active in *E. coli* [23, 24]. Notably, the expression of SOS-controlled DinQ and TisB proteins lead to a decrease in membrane potential [25] [26]. Furthermore, SOS-dependent induction of TisB expression significantly increases the level of persister cells resistant to CIP [27]. It has been hypothesised that subinhibitory QAC-concentrations may cause oxidative stress, which triggers the SOS response [28]. Hong et al. (2012) suggested that this external stress caused by a disinfectant (i.e. hydrogen peroxide) converts the bacterial population into persister cells [29]. Because exposure to BKC decreases the membrane potential, dormancy could be the main mechanism for reducing the sensitivity of bacterial cells to CIP when exposed to BKC.

Previous research clearly indicates a relation between the colony-forming ability and membrane potential. More than two decades ago it was suggested that DiBAC_4_ (3) fluorescence (i.e. loss of membrane potential) relates more closely to the loss of colony-forming ability than PI fluorescence (i.e. loss of membrane integrity) [21]. Our data appears to contradict this, with both an increase in plate counts and in the intermediate subpopulation of DiBAC_4_ (3)^+^/PI^−^ cells. However, it remains unclear if these intermediate bacteria lose their pumping activity due to the decrease in membrane potential [30] or to dormancy. Importantly, our differential bacterial count results indicate that BKC exposure triggered a CIP-resistance mechanism in the disinfectant-exposed *E. coli* isolates. A stress-induced efflux in *E. coli* through activation or overexpression of genes in the *mar* regulon has been widely described [31–33]. More specifically, Langsrud et al. (2004) and Bore et al. (2007) implied that BKC exposure could initiate a general stress reaction in *E. coli* followed by enhanced efflux activity of the BKC-adapted bacterial cells [34, 35]. In addition, CIP has been reported to be exported by both efflux pump systems of the resistance nodulation family (AcrAB-TolC efflux system) as well as the major facilitator superfamily (MdfA) in *E. coli* and furthermore to facilitate the extrusion of BKC [36, 37]. In summary, since multidrug efflux pumps have been described in *E. coli* extruding both CIP and BKC, and the bacterial counts following CIP treatment were significantly higher after exposure to a subinhibitory BKC concentration, it is hypothesised that this disinfectant exposure altered the efflux of CIP. The occurrence of an increased mutation frequency induced by exposure to subinhibitory BKC concentrations could be a second explanation for the observed higher plate counts. Ebrahimi et al. (2017) demonstrated that exposure of *E. coli* to subinhibitory BKC could select mutants with a significantly decreased susceptibility to both BKC and CIP [38]. Physiological conditions such as bacterial stress may regulate the mutation frequency in bacteria as reviewed by Martinez and Baquero (2000) and Poole (2012) [39, 40]. Therefore, so-called adaptive mutants could be the result of stress-induced mutagenesis [41] caused by BKC and may lead to several resistance mechanisms such as over-expression of efflux pumps [33].

A second key finding which partially confirmed the growth-dependent method data was the high viability of the CIP-susceptible isolates (> 50% live subpopulation) for all CIP concentrations as shown by FCM. These unexpectedly high levels of survival upon CIP treatment could be explained by a CIP-induced persister formation as a result of a SOS response mechanism [24, 27]. Comparing both these methods, the percentages of live bacteria (FCM) were systematically higher than the number of CFU/mL (plate counts) for all CIP concentrations, which demonstrates that the *E. coli* isolates gradually did lose their culturability following antibiotic treatment.

A third key finding was the similar number of CFU/mL for the BKC-exposed *E. coli* isolates, while FCM results showed a clear shift from live to intermediate and dead subpopulations when exposed to subinhibitory BKC concentrations. The relatively high percentage of the dead subpopulation after subinhibitory BKC exposure could not be observed when the *E. coli* isolates were subsequently treated with CIP at any of the evaluated concentrations. More specifically, these CIP-treated *E. coli* isolates showed a clear shift from the live to the intermediate bacterial subpopulation following exposure to subinhibitory QAC concentrations versus non-QAC exposed CIP-treated bacteria.

This confirms the added value of the growth-independent FCM, a technique that can provide complementary information on the viability state compared to the microbiological plate technique quantifying only culturable bacteria as previously reported [42, 43].

## Conclusions

In conclusion, our study demonstrated that exposure to subinhibitory concentrations of BKC increases the number of culturable *E. coli* bacteria after CIP treatment. Our results also suggest a BKC-dependent onset of membrane damage and loss of membrane potential. The bacteria with a damaged membrane are not dead, but rather enter a dormant state that consists of both non-culturable and potentially culturable bacteria. Further research is needed to elucidate if the induced injury of this intermediate population is potentially reversible and to clarify if multidrug efflux systems are responsible for the increased culturability after BKC exposure. In addition, these results should be supported by observations more relevant to the field, as commercial disinfectants are composed of combinations of active disinfectant components (e.g. BKC and glutaraldehyde). Nevertheless, this study highlights the concerns about the presence of subinhibitory BKC or disinfectant concentrations in agricultural settings and consequently the need for standardised cleaning and disinfection protocols.

## Methods

### Biocides, antibiotics and dyes

The antimicrobial components ciprofloxacin (CIP, PHR1044) and benzalkonium chloride (BKC, 12060) were obtained from Sigma Aldrich (Saint Louis, MO, USA). Stock concentrations of 10 mg/mL CIP were prepared in distilled water, filtered through a 0.2 μm filter and aliquots were stored at − 20 °C. Working concentrations of CIP were based on the epidemiologic cutoff (ECOFF) value of *E. coli* defined by EUCAST. CIP concentrations of 0.064 mg/L (1 x ECOFF), 0.640 mg/L (10 x ECOFF) and 6.400 mg/L (100 x ECOFF) were diluted in cation-adjusted Mueller-Hinton broth with TES buffer (CAMHB, Thermo Scientific, YT3462).

#### Subinhibitory concentration

For exposure to BKC a subinhibitory concentration of 0.00675 g/L BKC in Tryptone Soya Broth (TSB, Oxoid, CM0129) was used [44]. This was based on a preliminary dose-effect experiment in which this concentration did not affect the number of CFU per mL and the antibiotic (CIP) effect after BKC exposure could still be evaluated, shown in detail in Table 2.

**Table 2:** Viability (%) and plate count results of the preliminary dose effect experiment with and without exposure to benzalkonium chloride

***E. coli*** isolate			BKC concentration (g/L)			
			**0.0000**	**0.00675**	**0.01350**	**0.02700**
KL6	**Viability (%)**	Live	97.15	71.38	46.49	23.16
		Intermediate	0.61	21.57	22.59	75.39
		Dead	2.06	6.94	30.89	1.43
	**Plate counts (log CFU/mL)**		8.77	8.61	8.08	> 4.00
KQ26	**Viability (%)**	Live	97.64	54.73	35.67	0.34
		Intermediate	0.35	8.32	14.47	98.20
		Dead	1.60	36.45	49.70	1.41
	**Plate counts (log CFU/mL)**		9.15	8.81	8.16	4.71
VA57	**Viability (%)**	Live	92.13	84.33	36.22	3.64
		Intermediate	1.44	2.04	12.02	94.33
		Dead	5.46	13.44	51.62	1.94
	**Plate counts (log CFU/mL)**		8.97	8.77	7.95	6.02
ATCC 10536	**Viability (%)**	Live	96.97	78.26	45.63	0.87
		Intermediate	0.88	8.64	22.47	98.36
		Dead	1.93	12.58	31.70	0.75
	**Plate counts (log CFU/mL)**		8.98	7.69	7.23	3.87

*CFU* colony forming units, *BKC* benzalkoniumchloride

The first dye used in this study was bis-(1,3-dibutylbarbituric acid) trimethine oxonol (DiBAC_4_ (3), Invitrogen, Molecular Probes, B438, Carlsbad, CA, USA) (excitation 490 nm and emission 516 nm) to measure the membrane potential. The second dye used was Propidium Iodide (PI, Sigma Aldrich, 79,214) (excitation 535 nm and emission 615 nm) to measure the membrane integrity. A stock solution (250 μmol/L) of DiBAC_4_ (3) was made up in Dimethyl Sulfoxide (DMSO) and kept in the dark at − 20 °C. PI was supplied by the manufacturer as a solution of 1.0 mg/mL in distilled water and stored at 4 °C in the dark.

### Strain selection and MIC determination

Three *Escherichia coli* (*E. coli*) field isolates obtained from a previous study [44] and *E. coli* ATCC 105836, a reference strain used in the EN 1276 suspension test for the evaluation of the bactericidal efficacy of chemical disinfectants, were selected. Isolate characteristics are provided in Table 1. Field isolates with a minimal inhibitory concentration (MIC) for CIP just above and below the epidemiological cutoff value (ECOFF) were used. The ECOFF value separates micro-organisms with and without acquired resistance. Micro-organisms without acquired resistance mechanisms are considered as ‘wild-type (WT)’. In this manuscript, bacterial strains were considered either as ‘resistant’ (non-wild type WT) or as ‘susceptible’ (WT). To determine the inter-assay variation *E. coli* ATCC 25922 was used as reference in every experiment.

### Strain preparation

*E. coli* isolates from frozen glycerol stock were streaked on fresh Plate Count Agar plates (PCA, Oxoid, CM0325, Basingstoke, Hampshire, England) and incubated overnight at 37 °C. Per agar plate, 1 colony was picked to inoculate 10 mL of Tryptone Soya Broth (TSB, Oxoid, CM0129) and grown at 37 °C for 16 h to obtain fresh liquid cultures. Subsequently, liquid cultures were centrifuged in Eppendorf tubes at 10,000 *g* for 5 min at 4 °C. The supernatant was discarded and the pellet was resuspended in 0.9% NaCl to obtain a bacterial culture for the biocide and antibiotic treatments with an optical density at 600 nm (OD600) corresponding to a viable count of 1–5 × 10^8^ CFU/mL to ensure a final concentration in the range between 2 × 10^4^ and 2 × 10^7^ for an accurate flow cytometric analysis.

### Biocide treatment procedure

In a Cellstar 15 mL tube (Greiner bio-one, Vilvoorde, Belgium) 5 mL of the BKC solution and 5 mL of the bacterial cell suspension were added, resulting in a total volume of 10 mL at a subinhibitory concentration of 0.00675 g/L BKC. As positive control (= control for live cells) the BKC solution was replaced by 5 mL TSB. As blank 10 mL of TSB was used. All tubes were incubated for 24 h at 37 °C.

#### Washing and dilution

After incubation, the cell suspension was sonicated for 5 min to prevent bacterial aggregates and homogenisation using a vortex. Subsequently, 1 mL of the suspension was transferred to an Eppendorf tube and centrifuged for 5 min at 10,000 *g* at 4 °C. The supernatant was gently discarded after which 1000 μL of cold 0.9% NaCl was added to wash the pellet. After repeating the last step, tubes were sonicated and diluted by transferring 50 μL of the cell suspension in a new Eppendorf tube filled with 450 μL 0.9% NaCl. As control for live cells, 50 μL of the cell suspension without BKC and 450 μL 0.9% of NaCl were added in an Eppendorf tube in duplicate after which one of the duplicates was heated for 15 min at 100 °C to use as control for cell death.

### Antibiotic treatment procedure

A mixture of 2.5 mL antibiotic solution and 2.5 mL of the cell suspension was added to a Cellstar tube (Greiner bio-one) resulting in a total volume of 5 mL at a concentration of 0.064 mg/L CIP, 0.640 mg/L CIP or 6.400 mg/L CIP. As control for live cells, the volumes with antibiotics were replaced by 2.5 mL CAMHB. As blank, 5 mL of CAMHB was used. All tubes were incubated for 24 h at 37 °C.

#### Washing and dilution

After incubation, the same washing and dilution steps were applied as described in the biocide treatment except for the wash solution, which was replaced by CAMHB.

### Antibiotic treatment procedure after biocide exposure

Samples exposed to biocides as explained above were washed twice, after which 1 mL of the washed biocide-exposed cell suspension and 1 mL of the antibiotic solution were incubated for 24 h at 37 °C.

#### Washing and dilution

After incubation, the same protocol to wash and dilute the cell suspension was used as for the antibiotic treatment.

### Plate count analysis

After dilution, the number of CFU/mL of the non-stained cell suspension was assessed by spotting six times 20 μL with a multipipettor (Thermofisher Scientific, 2–20 μL) on PCA. Plates were incubated overnight at 37 °C. The lower limit for enumeration was 0.9 log CFU/mL.

### Flow cytometric double staining procedure

From the Eppendorf tubes containing 500 μL of treated cell suspensions, 250 μL was transferred to a 96-well plate to measure autofluorescence. The remaining 250 μL of cell suspension were stained by adding 2 μL DiBAC_4_ (3) and 2.5 μL PI to each Eppendorf tube. After 20 min of incubation at 37 °C, the stained suspension was also transferred to the 96-well plate and immediately analysed via FCM (CytoFLEX, Beckman Coulter).

### Flow cytometric analysis

To analyse the membrane potential and membrane integrity of the bacterial cells, blue laser light (488 nm, 50 mW) of the CytoFLEX (Beckman Coulter) was used for the excitation of DiBAC_4_ (3) and PI, respectively. Fluorescence was detected with the green emission filter for DiBAC_4_ (3) (525/40 nm) and the red emission filter for PI (690/50 nm). Samples were run at a flow rate of 10 μL/min. Flow cytometric analysis was based on the number of events (10,000 events in a gated population on a dot plot FS vs SS excluding debris and doublets) in order to estimate the subpopulations (%). Data analysis was performed in CytExpert (Version 2.0, Beckman Coulter) and Microsoft Excel (Microsoft Office 2016). All data are the result of experiments done in triplicate.

### Flow cytometric gating strategy

The FSC-A/SSC-A dot plots of each isolate were used to define the total bacterial population. Via the SSC-A/SSC-H dot plot, doublets and debris were excluded (> 90% single cells) shown in Figure S2 (see Additional File 5). Via the contour and dot plots, three different subpopulations (i.e., live, intermediate and dead bacteria) could be defined and were delimited using the ‘polygon’ tool in the DIBAC_4_ (3)/PI plots. Isolated dots outside the polygon were not included in the analysis.

### Statistical analysis

Statistical analyses were performed using the Statistical Analysis System software (SAS, version 9.4, SAS Institute Inc., Cary, NC, USA). A histogram and Q-Q plot were made of the data obtained to characterise the distribution of the variables for both antibiotics.

In order to evaluate the effect of BKC exposure on the culturability (plate counts in log CFU/mL) and viability (live and intermediate subpopulations) of the *E. coli* isolates after antibiotic treatment, a linear regression model was fitted to the data. Logarithmically transformed plate counts followed a normal distribution. Flow cytometric data of the viability percentages of live, intermediate and dead bacteria were arcsine-transformed to obtain normal distributions. Univariate linear regression was performed with the antibiotic resistance profile (resistant/susceptible), antibiotic treatment concentration (0.064 mg/L, 0.640 mg/L and 6.400 mg/L CIP), BKC treatment (no BKC exposure/BKC exposure) and isolate origin (poultry/pork/reference strain) as categorical independent variables. Variables with a *P*-value ≤0.2 in the univariable model were added to the multivariable regression model. Before performing multivariable linear regression, independent variables were assessed for two-way correlations using Spearman’s correlation test. Whenever the correlation coefficient was above 0.6, only one of the two variables was retained and added to the multivariable model. A backward stepwise elimination was performed to determine the final statistical multivariable model, starting with the global model (independent variables: BKC exposure, origin, CIP treatment and antibiotic resistance profile) then all non-significant variables were removed. *P*-values ≤0.05 were considered as significant.

## Supplementary information

**Additional file 1: Table S1.** Associations between the variables of the experiment and plate counts (log CFU/mL) after ciprofloxacin treatment.

**Additional file 2: Table S2.** Viability (%) results of both live and dead controls of each *Escherichia coli* isolate, expressed as mean +/− standard deviation.

**Additional file 3: Figure S1.** Illustrative flow cytometric DIBAC_4_ (3) (FITC-A)/PI (PC5.5A) dot plots representing a live and dead control.

**Additional file 4: Table S3.** Associations between the variables of the experiment and the viability of the live and intermediate *Escherichia coli* isolates’ subpopulations (%) after ciprofloxacin treatment.

**Additional file 5: Figure S2.** Example of a FSC-A/SSC-A dot plots to define the total bacterial population (A) and a SSC-A/SSC-H dot plot to exclude doublets and debris (B).

## Data Availability

The datasets used and analysed during the current study are available from the corresponding author on reasonable request.

## Abbreviations

BKC: Benzalkoniumchloride
C&D: Cleaning and disinfection
CAMHB: Cation-adjusted Mueller-Hinton broth
CFU: Colony forming units
CIP: Ciprofloxacin
DiBAC_4_ (3): Bis-(1,3-dibutylbarbituric acid) trimethine oxonol
DMSO: Dimethyl Sulfoxide
*E. coli*: *Escherichia coli*
ECOFF: Epidemiological cutoff
FCM: Flow cytometry
MIC: Minimum Inhibitory Concentration
PCA: Plate Count Agar
PI: Propidium Iodide
QAC: Quaternary ammonium compound
TSB: Trypton Soya Broth
VBNC: Viable but non-culturable
WT: Wild-type
