# Supplementary material for: Effect of subinhibitory exposure to quaternary ammonium compounds on the ciprofloxacin susceptibility of Escherichia coli strains in animal husbandry
Source: BMC Microbiol. 2020 Jun 11;20:155. doi: 10.1186/s12866-020-01818-3 (PMC7291530; doi:10.1186/s12866-020-01818-3)
Supplement: Supplementary file 1 — Additional file 1: Table S1. Associations between the variables of the experiment and plate counts (log CFU/mL) after ciprofloxacin treatment. [file 12866_2020_1818_MOESM1_ESM.docx]

Table S1:

|  | Log CFU/mL | | | | | | | | | | | |  |
| --- | --- | --- | --- | --- | --- | --- | --- | --- | --- | --- | --- | --- | --- |
|  | Univariable analysis | | | | | |  | Multivariable analysis | | | | |  |
| Variable | β-coefficient | | SE | | P-value | |  | β-coefficient | | SE | P-value | |  |
|  |  | |  | |  | |  |  | |  |  | |  |
| BKC exposure |  | |  | | < 0.001 | |  |  | |  | < 0.001 | |  |
| without | ref. | | ref. | |  | |  | ref. | | ref. |  | |  |
| with | 2.102 | | 0.144 | |  | |  | 2.102 | | 0.116 |  | |  |
| CIP treatment (mg/L) |  | |  | | < 0.001 | |  |  | |  | < 0.001 | |  |
| 0.064 | ref. | | ref. | |  | |  | ref. | | ref. |  | |  |
| 0.640 | -1.964 | | 0.176 | |  | |  | -1.964 | | 0.142 |  | |  |
| 6.400 | -2.538 | | 0.176 | |  | |  | -2.538 | | 0.142 |  | |  |
| CIP resistance profile |  | |  | | 0.0038 | |  |  | |  | < 0.001 | |  |
| Susceptible | ref. | | ref. | |  | |  | ref. | | ref. |  | |  |
| Resistant | 1.357 | | 0.453 | |  | |  | 0.997 | | 0.163 |  | |  |
| Origin |  | |  | | 0.006 | |  |  | |  | < 0.001 | |  |
| Reference strain | ref. | | ref. | |  | |  | ref. | | ref. |  | |  |
| Poultry | 1.586 | | 0.176 | |  | |  | 1.086 | | 0.164 |  | |  |
| Pork | 1.093 | | 0.203 | |  | |  | 1.093 | | 0.164 |  | |  |
|  |  |  | |  | |  |  | |  | | |  | |

CFU: colony forming units, BKC: benzalkonium chloride, CIP: ciprofloxacin, SE: standard error of the mean, ref: reference. Numbers in bold correspond to significant P-values
