# Supplementary material for: Effect of subinhibitory exposure to quaternary ammonium compounds on the ciprofloxacin susceptibility of Escherichia coli strains in animal husbandry
Source: BMC Microbiol. 2020 Jun 11;20:155. doi: 10.1186/s12866-020-01818-3 (PMC7291530; doi:10.1186/s12866-020-01818-3)
Supplement: Supplementary file 2 — Additional file 2: Table S2. Viability (%) results of both live and dead controls of each Escherichia coli isolate, expressed as mean +/− standard deviation. [file 12866_2020_1818_MOESM2_ESM.docx]

Table S2:

| ***E. coli* isolate** | **Control** | **Subpopulation** | **Viability (%)** | | |
| --- | --- | --- | --- | --- | --- |
| KL6 | Live | Live | 98.44 | ± | 0.51 |
|  |  | Intermediate | 0.35 | ± | 0.17 |
|  |  | Dead | 0.17 | ± | 0.04 |
|  | Dead | Live | 0.55 | ± | 0.15 |
|  |  | Intermediate | 0.16 | ± | 0.07 |
|  |  | Dead | 99.22 | ± | 0.25 |
| KQ26 | Live | Live | 97.03 | ± | 0.30 |
|  |  | Intermediate | 0.95 | ± | 0.18 |
|  |  | Dead | 1.62 | ± | 0.28 |
|  | Dead | Live | 0.55 | ± | 0.02 |
|  |  | Intermediate | 0.46 | ± | 0.09 |
|  |  | Dead | 98.90 | ± | 0.10 |
| VA57 | Live | Live | 93.97 | ± | 0.30 |
|  |  | Intermediate | 1.37 | ± | 0.02 |
|  |  | Dead | 3.93 | ± | 0.31 |
|  | Dead | Live | 0.34 | ± | 0.36 |
|  |  | Intermediate | 0.32 | ± | 0.11 |
|  |  | Dead | 98.66 | ± | 1.25 |
| ATCC 10536 | Live | Live | 88.62 | ± | 2.29 |
|  |  | Intermediate | 2.74 | ± | 0.22 |
|  |  | Dead | 7.89 | ± | 2.09 |
|  | Dead | Live | 1.37 | ± | 0.32 |
|  |  | Intermediate | 2.74 | ± | 0.22 |
|  |  | Dead | 97.50 | ± | 0.29 |
