# Supplementary figures and images for: Effect of subinhibitory exposure to quaternary ammonium compounds on the ciprofloxacin susceptibility of Escherichia coli strains in animal husbandry
Source: BMC Microbiol. 2020 Jun 11;20:155. doi: 10.1186/s12866-020-01818-3 (PMC7291530; doi:10.1186/s12866-020-01818-3)

Figure S1:


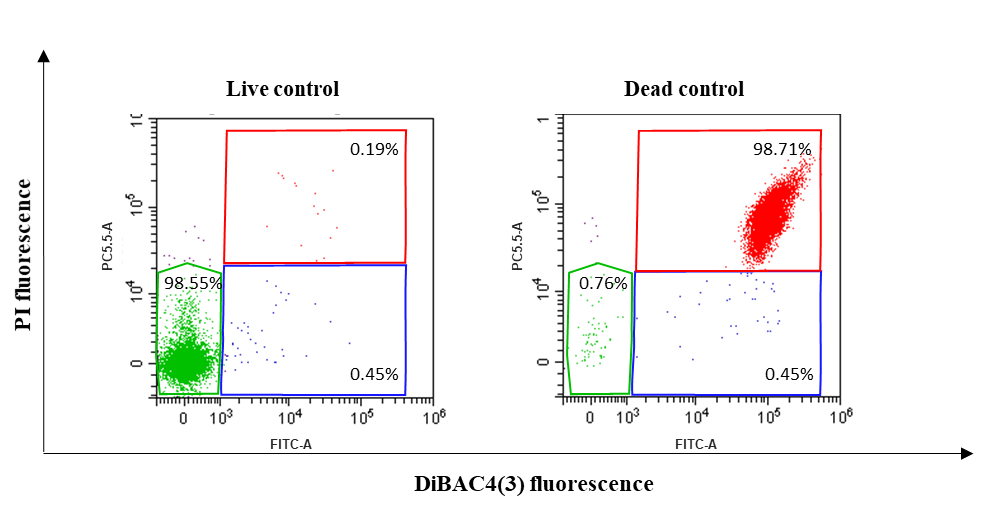

Supplement: Supplementary file 3 — Additional file 3: Figure S1. Illustrative flow cytometric DIBAC4 (3) (FITC-A)/PI (PC5.5A) dot plots representing a live and dead control. [file 12866_2020_1818_MOESM3_ESM.docx]

Figure S2:


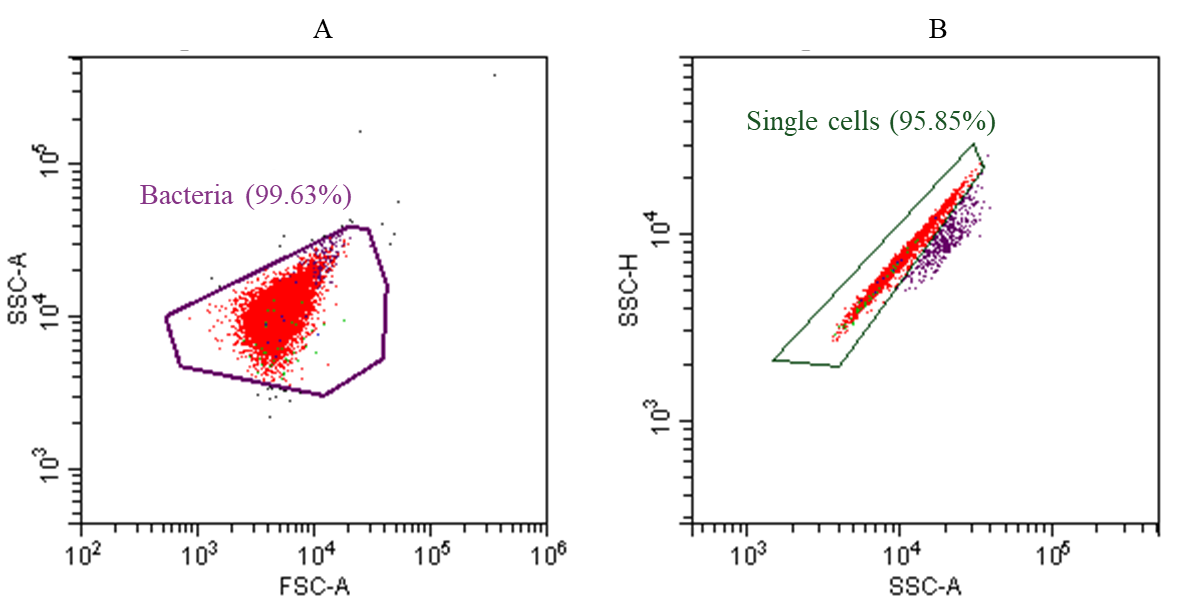

Supplement: Supplementary file 5 — Additional file 5: Figure S2. Example of a FSC-A/SSC-A dot plots to define the total bacterial population (A) and a SSC-A/SSC-H dot plot to exclude doublets and debris (B). [file 12866_2020_1818_MOESM5_ESM.docx]
