# Supplementary material for: Effect of subinhibitory exposure to quaternary ammonium compounds on the ciprofloxacin susceptibility of Escherichia coli strains in animal husbandry
Source: BMC Microbiol. 2020 Jun 11;20:155. doi: 10.1186/s12866-020-01818-3 (PMC7291530; doi:10.1186/s12866-020-01818-3)
Supplement: Supplementary file 4 — Additional file 4: Table S3. Associations between the variables of the experiment and the viability of the live and intermediate Escherichia coli isolates’ subpopulations (%) after ciprofloxacin treatment. [file 12866_2020_1818_MOESM4_ESM.docx]

Table S3:

|  | Live | | | | | | | | | | | | | | |  | | |  | | Intermediate | | | | | | | | | | | | | | | | |  |  |  |
| --- | --- | --- | --- | --- | --- | --- | --- | --- | --- | --- | --- | --- | --- | --- | --- | --- | --- | --- | --- | --- | --- | --- | --- | --- | --- | --- | --- | --- | --- | --- | --- | --- | --- | --- | --- | --- | --- | --- | --- | --- |
|  | Univariable analysis | | | | | |  | Multivariable analysis | | | | | | | |  | | Univariable analysis | | | | | | | | | | | Multivariable analysis | | | | | | | | |  |  |  |
| Variable | β-coefficient | | SE | | | P-value |  | | β-coefficient | | | SE | | | P-value |  | | | β-coefficient | | | | SE | | | P-value | | | |  | | β-coefficient | | | SE | | P-value |  |  |  |
| BKC exposure |  | |  | | | 0.072 |  | | | | |  | | | 0.035 |  | | |  | | | |  | | | 0.022 | | | |  | | |  | |  | | 0.007 |  |  |  |
| Without | Ref. | |  | | |  | Ref. | | | | |  | | |  |  | | | Ref. | | | |  | | |  | | | |  | | Ref. | |  | | |  |  |  |  |
| With | -0.085 | | 0.046 | | |  | -0.085 | | | | | 0.039 | | |  |  | 0.110 | | | | | | 0.047 | |  | | |  | | | | 0.110 | | 0.039 | | |  | |  | |
| CIP treatment (mg/L) |  | |  | | | 0.026 |  | | | | |  | | | 0.009 |  | |  | | | | |  | | | 0.010 | | | |  | |  | |  | | | <0.001 |  |  |  |
| 0.064 | Ref | |  | | |  | Ref. | | | | |  | | |  |  | Ref | | | | | |  | |  | | |  | | | | Ref. | |  | | |  | | |  |
| 0.640 | -0.092 | | 0.056 | | |  | -0.092 | | | | | 0.048 | | |  |  | 0.096 | | | | | | 0.056 | |  | | |  | | | | 0.096 | | 0.049 | | |  | | |  |
| 6.400 | -0.153 | | 0.056 | | |  | -0.153 | | | | | 0.048 | | |  |  | 0.178 | | | | | | 0.056 | |  | | |  | | | | 0.178 | | 0.049 | | |  | | |  |
| CIP resistance profile |  | |  | | | 0.039 |  | | | | |  | | |  |  |  | | | | | |  | | 0.076 | | | | | |  |  | |  | | |  | | |  |
| Susceptible | Ref. | |  | | |  |  | | | | |  | | |  |  | Ref. | | | | | |  | |  | | |  | | | |  | |  | | |  | | |  |
| Resistant | 0.112 | | 0.053 | | |  |  | | | | |  | | |  |  | -0.099 | | | | | | 0.055 | |  | | |  | | | |  | |  | | |  | | |  |
| Origin |  | |  | | | 0.001 |  | | | | |  | | | 0.001 |  | |  | | | | |  | | | 0.001 | | | |  | |  | |  | | | 0.002 |  |  |  |
| Reference strain | Ref. | |  | | |  | Ref. | | | | |  | | |  |  | Ref. | | | | | |  | |  | | |  | | | |  | |  | | |  | | |  |
| Poultry | 0.214 | | 0.052 | | |  | 0.214 | | | | | 0.048 | | |  |  | -0.211 | | | | | | 0.054 | |  | | |  | | | | -0.211 | | 0.049 | | |  | | |  |
| Pork | 0.093 | | 0.060 | | |  | 0.093 | | | | | 0.056 | | |  |  | -0.147 | | | | | | 0.062 | |  | | |  | | | | -0.147 | | 0.056 | | |  | | |  |
|  |  |  | |  |  | | | |  |  |  | |  |  | | | | | |  | |  | |  | | |  | | | | |  | | | |  | |  |  |  |

BKC: benzalkonium chloride, CIP: ciprofloxacin, SE: standard error of the mean, ref: reference. Numbers in bold correspond to significant P-value
